# Systemic MCP-1 Levels Derive Mainly From Injured Liver and Are Associated With Complications in Cirrhosis

**DOI:** 10.3389/fimmu.2020.00354

**Published:** 2020-03-11

**Authors:** Alexander Queck, Hannah Bode, Frank E. Uschner, Maximilian J. Brol, Christiana Graf, Martin Schulz, Christian Jansen, Michael Praktiknjo, Robert Schierwagen, Sabine Klein, Christian Trautwein, Hermann E. Wasmuth, Marie-Luise Berres, Jonel Trebicka, Jennifer Lehmann

**Affiliations:** ^1^Department of Internal Medicine 1, University Hospital, Goethe University, Frankfurt, Germany; ^2^Department of Internal Medicine 1, University Hospital, University Bonn, Bonn, Germany; ^3^Department of Internal Medicine III, RTWH Aachen, Aachen, Germany; ^4^European Foundation for the Study of Chronic Liver Failure, Barcelona, Spain; ^5^Institute of Clinical Research, Odense University Hospital, University of Southern Denmark, Odense, Denmark

**Keywords:** acute-on-chronic liver failure (ACLF), decompensated liver cirrhosis, inflammation, monocyte chemotactic protein 1 (MCP-1), transjugular intrahepatic portosystemic shunt (TIPS)

## Abstract

**Background and Aims:** Monocyte chemotactic protein-1 (MCP-1) is a potent chemoattractant for monocytes. It is involved in pathogenesis of several inflammatory diseases. Hepatic MCP-1 is a readout of macrophage activation. While inflammation is a major driver of liver disease progression, the origin and role of circulating MCP-1 as a biomarker remains unclear.

**Methods:** Hepatic CC-chemokine ligand 2 (*CCL2*) expression and F4/80 staining for Kupffer cells were measured and correlated in a mouse model of chronic liver disease (inhalative CCl_4_ for 7 weeks). Next, hepatic RNA levels of *CCL2* were measured in explanted livers of 39 patients after transplantation and correlated with severity of disease. Changes in MCP-1 were further evaluated in a rat model of experimental cirrhosis and acute-on-chronic liver failure (ACLF). Finally, we analyzed portal and hepatic vein levels of MCP-1 in patients receiving transjugular intrahepatic portosystemic shunt insertion for complications of portal hypertension.

**Results:** In this mouse model of fibrotic hepatitis, hepatic expression of *CCL2* (*P* = 0.009) and the amount of F4/80 positive cells in the liver (*P* < 0.001) significantly increased after induction of hepatitis by CCl_4_ compared to control animals. Moreover, strong correlation of hepatic *CCL2* expression and F4/80 positive cells were seen (*P* = 0.023). Furthermore, in human liver explants, hepatic transcription levels of *CCL2* correlated with the MELD score of the patients, and thus disease severity (*P* = 0.007). The experimental model of ACLF in rats revealed significantly higher levels of MCP-1 plasma (*P* = 0.028) and correlation of hepatic *CCL2* expression (*R* = 0.69, *P* = 0.003). Particularly, plasma MCP-1 levels did not correlate with peripheral blood monocyte *CCL2* expression. Finally, higher levels of MCP-1 were observed in the hepatic compared to the portal vein (*P* = 0.01) in patients receiving TIPS. Similarly, a positive correlation of MCP-1 with Child-Pugh score was observed (*P* = 0.018). Further, in the presence of ACLF, portal and hepatic vein levels of MCP-1 were significantly higher compared to patients without ACLF (both *P* = 0.039).

**Conclusion:** Circulating levels of MCP-1 mainly derive from the injured liver and are associated with severity of liver disease. Therefore, liver macrophages contribute significantly to disease progression. Circulating MCP-1 may reflect the extent of hepatic macrophage activation.

## Introduction

Unresolved hepatic inflammation is known to be a major driver of progression in liver disease (1, 2). Hereby, composition of resident and infiltrating monocyte derived macrophages plays a pivotal role in homeostasis of hepatic inflammation and development of fibrosis (3). The phenotype of M2-macrophages (alternative type) is involved in tissue repair and resolution of inflammation in liver disease, whereas M1 phenotype (classic type) leads to pro-inflammatory signaling (4). Thus, monocyte chemotactic protein 1 (MCP-1) recruits peripheral monocytes to the liver and supports takeover of M1 dominant phenotype in hepatic macrophages (5). Systemic inflammation is known to be the key mediator for the development of acute-on-chronic liver failure (ACLF), and an increase in leucocytes and C-reactive protein is strongly associated with the onset of ACLF (6). Nevertheless, activation of Kupffer cells and hepatic monocyte recruitment in ACLF suggest an important role of hepatic inflammation in ACLF development (7, 8). Mechanistically, bacterial translocation takes place in advanced liver disease with portal hypertension, resulting in consecutive inflammation and oxidative stress in the portal venous compartment (9, 10). Early diagnosis and prevention of ACLF is essential, since 28-day mortality rates of 30% and higher are reported (11). Therefore, we hypothesized that levels of MCP-1 in the portal vein and hepatic transcription of *CCL2* may be associated with severity of liver disease and complications of cirrhosis, including ACLF. To address this hypothesis, we measured MCP-1 transcription in explanted cirrhotic livers and MCP-1 levels in portal and hepatic venous blood from patients with decompensated cirrhosis at transjugular intrahepatic portosystemic shunt insertion (TIPS), and further confirmed our findings in animal models of cirrhosis and ACLF.

## Patients and Methods

### Patients, Animal Models, and Methods

Our study was undertaken to investigate the role of MCP-1 as a marker of liver disease progression, associated complications and ACLF. To this end, we performed two animal models of cirrhosis and ACLF, and included two patient cohorts (TIPS and liver transplantation).

#### Mouse Model of Toxic Liver Fibrosis

As previously described and published (12), male wild-type (WT, C57Bl6/J) mice (12 weeks old) were purchased (Charles River Laboratories Research Model and Services Germany, Sulzfeld, Germany). A total of 11 mice were used for this study. The animals were kept at 22°C with a 12:12-h day-night cycle in individually ventilated cages. Liver injury was induced by inhalative CCl_4_ exposure for seven weeks (once/week for the first four weeks, followed by intoxications twice/week for the next three weeks). Briefly, CCl_4_ was insufflated with a flow of 2 l/min for 1 min, the cage remaining closed for another minute, and CCl_4_ finally removed under the hood for 10 min. Water and chow were provided *ad libitum*. All animals intoxicated with CCl_4_ additionally received phenobarbital (0.33 g/l) via drinking water as an inducer of the cytochrome P-450 metabolic activity. Control age-matched untreated mice were used in the experiments. Before being euthanized, mice received ketaminexylazine anesthesia (100 mg ketamine/kg body weight and 10 mg xylazine/kg body weight) via intraperitoneal injection. Liver samples were fixed in formaldehyde (4%) and subsequently embedded in paraffin. All experiments were performed in accordance with the German Animal Welfare Act and the guidelines of the animal care facility of the University Hospital, Bonn (Haus für Experimentelle Therapie, University Hospital Bonn, Germany), and were approved by the North Rhine-Westphalian State Agency for Nature, Environment, and Consumer Protection (LANUV; file reference LANUV NRW, 84–02.04.2015.A491).

##### F4/80 staining of liver tissue

Stainings were performed on paraffin slides (2–3 μm) for F4/80 immunohistochemistry (IHC). F4/80 IHC was analyzed by counting positive stained cells in high power fields captured at a 200x magnification. A minimum of 12 high power fields were used for analysis.

#### Patient Cohort 1 (Liver Transplantation)

In patient cohort 1 (liver explants), 39 cirrhotic patients undergoing liver transplantation were included for measurement of hepatic mRNA expression of MCP-1. These patients were enrolled between 1999 and 2005 at the Department of Internal Medicine I, University of Bonn, Germany, as previously described (13). The study protocol was accepted and approved by the local ethics committee of the University of Bonn (029/13).

##### Quantitative PCR

Total RNA was isolated with ReliaPrep™ RNA Miniprep Systems (Promega, Madison, WI, USA) from shock-frozen liver tissue following the ReliaPrep™ RNA Cell Miniprep System protocol. Hereby, RNA concentration was measured spectrophotometrically at 260 nm. For each sample, 1 μg of total RNA was used. cDNA synthesis was performed by the ImProm-II Reverse Transcription System (Promega, Madison, WI, USA). Primers and probes for the housekeeping gene (18SrRNA) were provided by Thermo Fisher Scientific, Waltham, MA, USA, as a ready-to-use mix. Every sample underwent two DNase digestion steps to dispose of genomic DNA. Quantitative PCR (qPCR) was carried out using TaqMan gene expression assay for *CCL2* (assay ID Hs00234140_m1, Thermo Fisher Scientific, Waltham, MA, USA) according to the manufacturer's protocol on a 7300 Real-Time PCR System (Applied Biosystems, Foster City, CA, USA. The PCR reaction was performed in a 25-μL volume containing 12.5 μL of 2 × TaqMan-PCR master mix and 2 μL (equivalent to 67 ng of total RNA) of cDNA. The final concentrations were 100 nM for the primers and 200 nM for the probe. Firstly, measurement of cycle threshold values was performed (Ct). Secondly, adjustment with endogenous controls created the ΔCt value was performed. Matching the ΔCt value between study and control group created the ΔΔCt value. The final results of the liver samples were expressed as 2^−ΔΔCt^ and revealed the x-fold change of gene expression compared to the control group. Experiments were carried out in duplicates and results were normalized to 18S rRNA. Duplicate measurements were used as a method to control for loading error. If duplicates were far apart, we either measured once again or we disregarded the sample. Measurement of mRNA levels were performed as previously described (14–16).

#### Rat Model of Cirrhosis and ACLF

##### Induction of liver cirrhosis and ACLF

Male wildtype (WT) Sprague Dawley rats were used. The experiments were performed according to the guidelines and regulations approved by LANUV, the responsible committee for animal studies in North Rhine-Westphalia, Germany. All rats were placed in a controlled environment (12 h light/dark; temperature 22–24°C) and received water and standard rat feed (Ssniff, Soest, Germany) *ad libitum*. Bile duct ligation (BDL) was performed in male WT rats with an initial body weight (BW) of 180–200 g to induce cholestatic liver cirrhosis, as previously described (17). ACLF was induced twice via intraperitoneal injection (day 21 and day 25 after BDL) of 6.25 μg/kg bodyweight (BW) lipopolysaccharid (LPS; *E. coli* O55:B5, Sigma-Aldrich, St. Louis, USA). Untreated BDL and sham-operated rats served as controls.

##### Tissue and blood collection

At the end of the experiment, liver samples were harvested, snap-frozen and stored at −80°C. Peripheral blood was collected in EDTA tubes (Sarstedt, Nümbrecht, Germany) for isolation of peripheral blood mononuclear cells (PBMCs). PBMC were isolated by density gradient centrifugation using Pancoll (PAN-Biotec, Aidenbach, Germany), as previously described (Beyer M, Abdullah, Nat Immunol, 2016). Cells were suspended in RPMI 1640 media with 10% fetal calf serum and 10% dimethyl sulfoxide (Gibco, Carlsbad, USA) and stored at −80°C.

##### Transcriptome analysis

Transcriptome analysis was performed by PakLabs (Hennigsdorf, Germany) using the Agilent Microarray XS (Agilent Technologies, Santa Clara, USA). Briefly, Low Input QuickAmp Labeling Kit (Agilent Technologies, Santa Clara, USA) was used to create fluorescent complementary RNA (cRNA) followed by hybridization to microarrays using the Gene Expression Hybridization Kit (Agilent Technologies, Santa Clara, USA). Fluorescence signals were detected using SureScan Microarray Scanner (Agilent Technologies, Santa Clara, USA).

##### Assessment of peripheral blood MCP-1

Peripheral blood circulating MCP-1 in rodents was measured in 25 μl of plasma using a multiplexed bead-based immunoassay (Milliplex MAP Cytokine/Chemokine Magnetic Bead Panel) (Merck Millipore, Darmstadt, Germany) on a Luminex 100 Bioanalyzer (Luminex Corp., Austin, TX), as described previously (6). The readouts were analyzed with Milliplex Analyst software (Merck Millipore) and a five-parameter logistic regression model was used to calculate the concentration of each sample (pg/ml).

#### Patient Cohort 2 (TIPS)

Patient cohort 2 (TIPS) consisted of 18 patients with diagnosed liver cirrhosis and severe portal hypertension undergoing TIPS-insertion. Patients from this cohort were recruited between May 2000 and April 2003 at the Department of Internal Medicine I, University of Bonn, Germany. Patients older than 18 years, with clinical signs of liver cirrhosis and a multidisciplinary defined indication for TIPS insertion were included in our trial. Exclusion criteria were presence of systemic infection, hepatic encephalopathy (higher than grade I), bilirubinemia (higher than 5mg/dl), or arterial pulmonary hypertension. Once the right branch of the portal vein was cannulated, we harvested blood from the portal and the hepatic vein in EDTA tubes (*N* = 18) for analysis of MCP-1. All TIPS insertions were performed without general anesthesia. After collection of the blood, we centrifuged the samples at 3,000 revolutions per minute for 15 min at 4°C. Afterwards, plasma samples were stored at −80°C, as previously described (18, 19). All patients provided written consent to all procedures, as declared in the study protocol.

##### Assessment of circulating MCP-1 levels

Plasma concentrations of MCP-1 from the portal and the hepatic vein in humans were also measured in 25 μl of plasma using a multiplexed bead-based immunoassay (Milliplex MAP Cytokine/Chemokine Magnetic Bead Panel) (Merck Millipore, Darmstadt, Germany) on a Luminex 100 Bioanalyzer (Luminex Corp., Austin, TX), as described previously (6, 13, 20). The readouts were analyzed with Milliplex Analyst software (Merck Millipore) and a five-parameter logistic regression model was used to calculate the concentration of each sample (pg/ml).

## Statistical Analyses

GraphPad Prism 5 for Windows (GraphPad Software, Inc.) or BIAS^®^ for Windows were used for the performance of statistical analyses. Wilcoxon matched-pairs signed rank test was used for paired intra-individual comparisons, namely portal versus hepatic vein MCP-1 concentrations. Group differences of unrelated groups were assessed by the Mann-Whitney test. Independent associations of variables were assessed in linear regression models. After univariate analyses, multivariate analyses were performed for significant associations using a *P* < 0.1. Variables with a *P* > 0.1 were discharged from the model. Correlations were assessed using Spearmen/Kendall rang correlation. *P* < 0.05 were considered to be statistically significant.

## Results

### Hepatic Levels of MCP-1 Are Increased in a Toxic Model of Liver Disease in Mice

**Figure 1:**
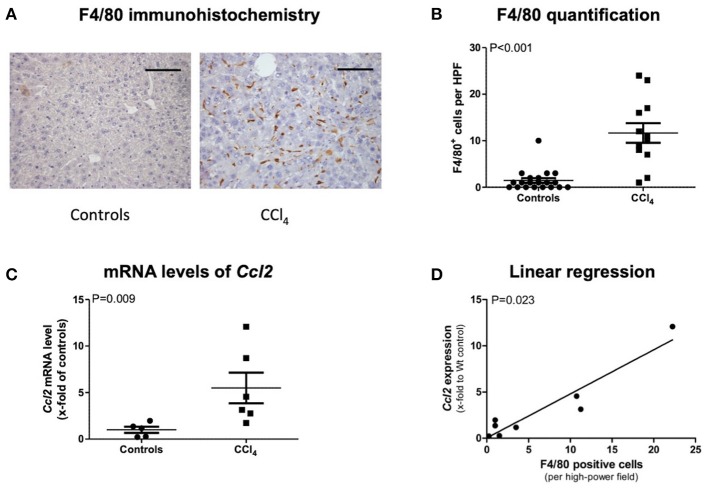
F4/80 immunohistochemistry staining **(A)** and its quantification **(B)**, transcript levels of *CCL2* in livers of CCl_4_ intoxicated mice **(C)**, and correlation of both **(D)**. The scale bar is 100μm **(A)**. Results are expressed as mean ± standard error of the mean (SEM); Mann–Whitney test **(B,C)**. Controls: *N* = 5, CCl4 group: *N* = 6. Spearman's rank correlation **(D)**.

In order to detect macrophages, we performed F4/80 immunohistochemistry staining in control and CCl_4_-intoxicated animals. Representative images show stronger F4/80 positivity in treated mice, which was confirmed via counting F4/80-positive cells (Figures 1A,B). Real-time PCR of *CCL2* revealed a statistically higher upregulation in CCl_4_-intoxicated animals compared to control mice (Figure 1C). Moreover, linear regression analysis showed a strong positive correlation between mRNA levels of *CCL2* and immunohistochemistry quantification of MCP-1 in our animal model (Figure 1D).

### Baseline Characteristic of Patient Cohort 1 (Liver Transplantation)

Patients undergoing liver transplantation (*N* = 39) were mainly male (*N* = 24; 62%), with a mean age of 47 years. Their mean MELD score was 15 (range 6–27). Most patients had Child Pugh score C (*N* = 20; 51%) and B (*N* = 16; 41%). Viral hepatitis was the main etiology of cirrhosis (*N* = 16; 41%), followed by alcohol (*N* = 12; 30%), and primary sclerosing cholangitis (*N* = 7; 18%). Ascites was present in 21 of the patients (54%). Fifteen patients (38%) had a history of gastrointestinal bleeding. Twenty percent of the patients had hepatorenal syndrome type 1 (*N* = 8), and another 70% (*N* = 27) had hepatorenal syndrome type 2. Fourteen patients (36%) were diagnosed with overt hepatic encephalopathy. All patient information is included in SI-Table 1.

### Hepatic *CCL2* Transcription Levels Were Associated With Severity of Disease at Liver Transplantation

**Figure 2:**
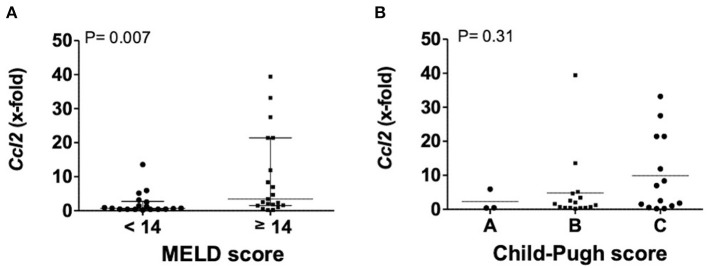
Transcription levels of *CCL2* in explanted livers in dependency of MELD **(A)** or Child-Pugh score **(B)**. Mann–Whitney test with *N* = 39 patients **(A)** and one way ANOVA with *N* = 33 patients **(B)**. Ccl2, (C-C motif) ligand 2; MELD, model for end stage liver disease, *P* < 0.05 were considered statistically significant.

Transcription levels of *CCL2* in explanted livers showed a correlation to severity of liver disease. Patients with a MELD score above 13 points had significantly higher levels of *CCL2*, compared to patients with a MELD score below 14 points (*P* = 0.0066). Patient stratification by Child-Pugh score also showed increasing levels from Child-Pugh level A to C, even if not statistically significant (Figure 2).

### MCP-1 Is Increased in Experimental ACLF and Correlates With Hepatic *CCL2* Expression

**Figure 3:**
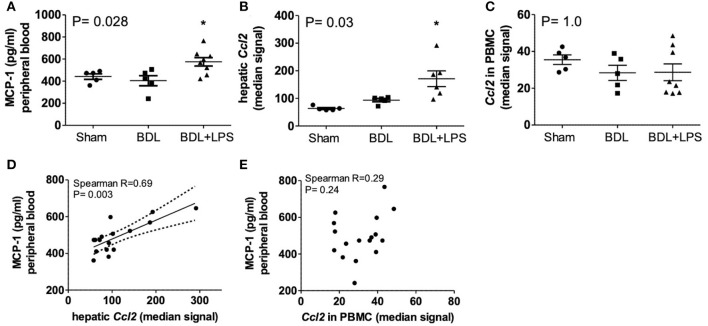
Levels of MCP-1 in peripheral blood **(A)**, expression of CCL2 in liver **(B)**, and PBMC **(C)** and correlation of blood MCP-1 with expression of CCL2 in liver **(D)** and PBMC **(E)** of healthy control, bile-duct ligated and ACLF rats. Mann–Whitney test and Spearman's rank correlation. Rats minimum *N* ≥ 5 per group. MCP-1, monocyte chemoattractant protein-1; *CCL2*; ACLF, acute on chronic liver failure; PBMC, peripheral blood mononuclear cells; BDL, bile duct ligation; LPS, lipopolysaccharide; *P* < 0.05 were considered statistically significant.

Bile duct ligation (BDL; 28 days) was performed in rats, and lipopolysaccharid (LPS; from *Escherichia coli*) was injected intraperitoneally (i.p.) on day 21 and day 25 after BDL for induction of ACLF. Circulating MCP-1 levels in peripheral blood were significantly increased in ACLF—(*P* = 0.028), compared to BDL—and healthy control animals. Furthermore, *CCL2* gene expression was highly upregulated in liver tissue (*P* = 0.03) but remained unchanged in isolated PBMCs from ACLF rats. Importantly, *CCL2* expression in liver tissue (*R* = 0.69; *P* = 0.003), but not in PBMC (*R* = 0.29; *P* = 0.24), correlated with the circulating MCP-1 levels in control, BDL and ACLF animals (Figure 3).

### Baseline Characteristics of Patient Cohort 2 (TIPS)

We included 18 patients undergoing TIPS insertion with a mean age of 59 years in the study. Refractory ascites (*N* = 9; 50%) was the major indication for TIPS insertion, followed by secondary prophylaxis of variceal bleeding (*N* = 6; 33%). In three patients (17%), indication was presence of esophageal variceal bleeding, as well as ascites. Most patients were admitted with alcohol-related liver cirrhosis (61%), followed by viral hepatitis (28%). Patients had a mean MELD score of 14 points (range 7–32), and most patients were classified as Child-Pugh B at the time of study inclusion. Presence of acute-on-chronic liver failure, calculated by the CLIF-C ACLF score, was seen in 5 out of 18 patients at TIPS insertion (28%). All ACLF patients presented with kidney failure−3 out of 8 patients with a CLIF-C organ failure (OF) score of seven points, and 5 out of 8 patients with a CLIF-C OF score of 8 points, respectively (SI-Table 2).

### Increased Hepatic Vein Levels of MCP-1 in Decompensated Cirrhosis at TIPS Insertion Are Correlated With Disease Severity

**Figure 4:**
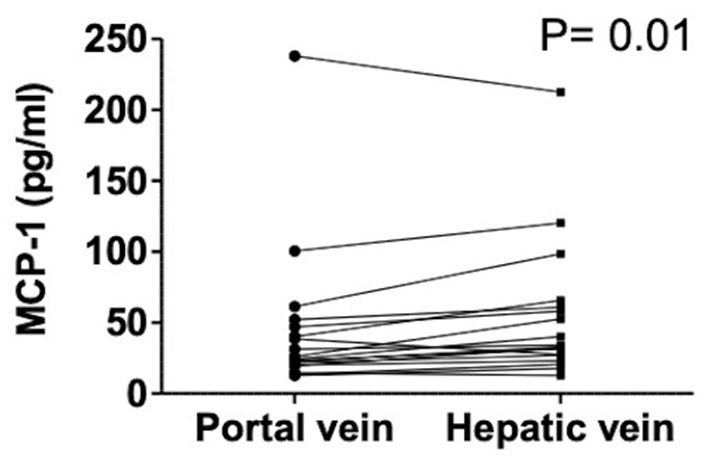
Levels of MCP-1 in portal and hepatic vein in decompensated cirrhosis. Wilcoxon matched-pairs signed rank test. Patients *N* = 18. MCP-1, monocyte chemoattractant protein-1; *P* < 0.05 were considered statistically significant.

**Figure 5:**
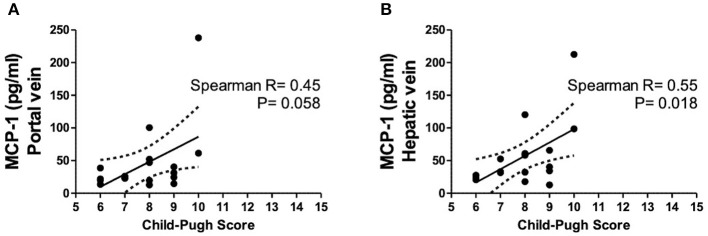
Levels of MCP-1 in portal **(A)** and hepatic vein **(B)** in dependency of Child-Pugh score. Spearman's rank correlation. Patients *N* = 8. MCP-1, monocyte chemoattractant protein-1; *P* < 0.05 were considered statistically significant.

**Figure 6:**
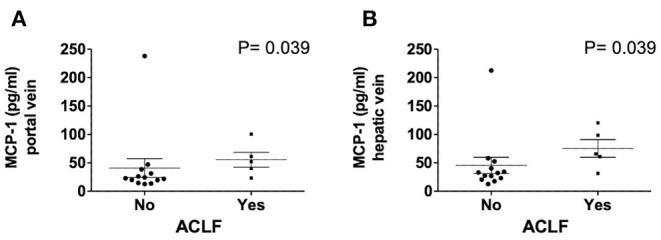
Levels of MCP-1 in portal **(A)** and hepatic vein **(B)** in dependency of ACLF. Mann–Whitney test. Patients *N* = 18. MCP-1, monocyte chemoattractant protein-1; ACLF, acute on chronic liver failure score; *P* < 0.05 were considered statistically significant.

Hepatic vein levels of MCP-1 were significantly higher compared to levels in the portal vein at TIPS insertion (*P* = 0.01) (Figure 4). Regression analyses revealed hepatic vein levels of MCP-1 associated with systemic levels of leucocytes (univariable *P* = 0.026, multivariable *P* = 0.04) and INR (univariable *P* = 0.01, multivariable *P* = 0.013). Furthermore, inverse association with systemic levels of albumin (univariable *P* = 0.002, multivariable *P* = 0.002) was observed (SI-Table 3). Moreover, portal vein levels of MCP-1 showed association with systemic levels of leucocytes (univariable *P* = 0.027, multivariable *P* = 0.036) and were inversely associated with albumin (univariable *P* = 0.004, multivariable *P* = 0.003) (SI-Table 4). Child-Pugh score positively correlated with MCP-1 levels in the hepatic vein (*R* = 0.55; *P* = 0.018) (Figure 5). Moreover, patients with ACLF had significantly higher levels of MCP-1 in the hepatic, as well as the portal vein (both *P* = 0.039) (Figure 6). Stratification of patients according to presence of gastrointestinal bleeding, ascites or hepatorenal syndrome did not show significant differences in portal or hepatic vein levels of MCP-1 (SI-Table 5).

## Discussion

The major finding of this study is that circulating MCP-1 is associated with severity of liver cirrhosis, and that it mainly derives from the diseased liver.

We could demonstrate that hepatic transcription of *CCL2* correlates with disease severity and cirrhosis-associated complications. Patients with higher levels of MCP-1 had a reduced survival, predicted by the MELD score and presented with more complications, evaluated by the Child-Pugh score, even in our rather small patient cohort.

To date, several animal studies have provided evidence that MCP-1 reflects monocyte recruitment and inflammation in liver disease (21, 22). Further, pharmacological inhibition of MCP-1 has been shown to result in reduced infiltration of macrophages into the liver and therefore, amelioration of steatohepatitis in rodent models (23).

We could observe that hepatic macrophages are possibly the main source of elevated systemic MCP-1 in decompensated cirrhosis, shown by increased hepatic transcription of *CCL2* in correlation with the number of hepatic macrophages (F4/80 staining) and increased MCP-1 levels in hepatic vein blood. This is of special interest since peripheral blood monocytes are known to be dysfunctional in decompensated cirrhosis with impaired anti-microbial ability (24). Therefore, liver-specific, inflammation-driven monocyte recruitment with tissue dependent MCP-1 activation and release could be a key mechanism of systemic inflammation.

Since the portal vein contains a unique immune composition and is known to be influenced by portal hypertension and bacterial translocation (10, 25), relatively higher MCP-1 levels in the hepatic vein reflect an overwhelming hepatic production with consecutive maintenance in monocyte recruitment and pro-inflammatory signaling. Furthermore, patients with acute-on-chronic liver failure, a syndrome closely associated with systemic inflammation and devastating mortality (26), had higher MCP-1 levels compared to patients without ACLF. This could be further confirmed in our rodent model of experimental cirrhosis and ACLF. Thus, ACLF induction led to an upregulation of *CCL2* expression in hepatic tissue, and consequently led to increased MCP-1 plasma levels in ACLF rats. Importantly, our data suggest that changes in MCP-1 plasma level are not associated with PBMC, but rather with diseased liver tissue, since circulating MCP-1 is strongly correlated with hepatic *CCL2* expression, but not with *CCL2* in PBMCs. The positive correlation of MCP-1 to the presence of ACLF further emphasizes the clinical relevance of monocyte recruitment and inflammation in cirrhosis. Interestingly, immune dysfunction in progression of cirrhosis was seen to be disease stage dependent. In a recently published study, patients with advanced, but compensated cirrhosis showed overwhelmingly pro-inflammatory signaling, while patients with ACLF presented signs of immune paralysis with a decrease of several pro-inflammatory cytokines in peripheral blood (27). In our study, presence of sepsis or acute infection are contraindications for TIPS insertion, whereas ACLF correlation to elevated MCP-1 levels in portal and hepatic vein revealed patients with subclinical, but immunological relevant inflammation. This possibly underlines the role of MCP-1 as a biomarker for early prediction of cirrhotic patients with high morbidity and mortality risk. Finally, we found independent inverse correlation of MCP-1 levels in the portal and the hepatic vein and systemic albumin levels. This correlation may also be due to the fact that albumin binds MCP-1 (28, 29).

Our study has several limitations. All our patients are decompensated patients, and TIPS placement was performed in a relatively small cohort of patients. Access to hepatic blood compartments and hepatic tissue in patients with advanced liver disease is rare and difficult to access. Therefore, samples are not paired as this is not possible in clinical practice. Moreover, MCP-1 assessment in our liver samples was only performed on the level of gene expression, but not on the protein level. Furthermore, presence of ACLF was only documented for the TIPS cohort and not for the liver transplantation cohort.

In conclusion, circulating levels of MCP-1 mainly derive from the injured liver and are associated with severity of liver disease. Therefore, liver macrophages contribute significantly to disease progression, and circulating MCP-1 may reflect the extent of hepatic macrophage activation.

## Data Availability Statement

The raw data supporting the conclusions of this article will be made available by the authors, without undue reservation, to any qualified researcher.

## Ethics Statement

The studies involving human participants were reviewed and approved by the Ethics Committee of the University of Bonn. The patients/participants provided their written informed consent to participate in this study.

## Author Contributions

AQ, HB, JL, and JT contributed to the manuscript by planning and initiating the study. AQ, HB, CG, MS, MB, CJ, MP, RS, FU, SK, CT, HW, M-LB, JL, and JT collected the data. AQ, HB, FU, MB, M-LB, JT, and JL performed the statistics. AQ, HB, FU, M-LB, JT, and JL interpretated data. AQ, HB, FU, RS, M-LB, JL, and JT drafted the manuscript. All authors critically discussed, corrected, and reviewed the manuscript.

### Conflict of Interest

The authors declare that the research was conducted in the absence of any commercial or financial relationships that could be construed as a potential conflict of interest.
