# Supplementary material for: Systemic MCP-1 Levels Derive Mainly From Injured Liver and Are Associated With Complications in Cirrhosis
Source: Front Immunol. 2020 Mar 11;11:354. doi: 10.3389/fimmu.2020.00354 (PMC7078155; doi:10.3389/fimmu.2020.00354)
Supplement: Supplementary file 1 [file Data_Sheet_1.docx]

Supplementary Material

SI-Table 1: Baseline characteristics of patients undergoing liver transplantation

| **Variables** | | **Patients characteristics (N=39)** |
| --- | --- | --- |
| Age at transplantation, years; mean (sd) | | 47 (11) |
| Gender, male/female; N (%) | | 24 (62)/ 15 (38) |
| Etiology of cirrhosis | alcohol; N (%)  viral hepatitis; N (%)  PBC; N (%)  PSC; N (%)  Morbus Wilson; N (%)  Cryptogenic; N (%) | 12 (30)  16 (41)  2 (5)  7 (18)  1 (3)  1 (3) |
| Child-Pugh class, A/ B/ C; N (%) | | 3 (8) / 16 (41) / 20 (51) |
| MELD score; mean (range) | | 15 (6-27) |
| Esophageal varices, grade 0/ 1/ 2/ 3; N (%) | | 9 (23)/ 10 (26)/ 14 (36)/ 6 (15) |
| Ascites, none/ mild/ severe; N (%) | | 18 (46)/ 11 (28) / 10 (26) |
| Hepatorenal syndrome; no/ type 1/ type 2 | | 4 (10)/ 8 (20)/ 27 (70) |
| History of variceal bleeding, no/ esophageal/ gastric | | 24 (62)/ 13 (33)/ 2 (5) |
| Hepatic encephalopathy, no/ yes; N (%) | | 25 (64) /14 (36) |

PBC: primary biliary cirrhosis; PSC: primary sclerosing cholangitis; MELD: model of end-stage liver disease; sd: standard deviation.

SI-Table 2: Baseline characteristics of patients receiving TIPS

| **Variables** | | **Patient characteristics (N=18)** |
| --- | --- | --- |
| Age at TIPS insertion, years; mean (sd) | | 59.6 (8.9) |
| Gender, male/female; N (%) | | 10/8 (56/44) |
| Etiology of cirrhosis | alcohol; N (%)  viral hepatitis; N (%)  cryptogenic; N (%) | 11 (61)  5 (28)  2 (11) |
| Child-Pugh class, A/ B/ C; N (%) | | 4 (22)/ 12 (67)/ 2 (11) |
| MELD score; mean (range) | | 14 (7-32) |
| Esophageal varices, grade 0/ 1/ 2/ 3; N (%) | | 2 (12)/ 3 (16)/ 9 (50)/ 4 (22) |
| TIPS indication, bleeding/ ascites/ HRS/ bleeding and ascites; N (%) | | 6 (33)/ 9 (50)/ 0 (0)/ 3 (17) |
| Ascites, none/ mild/ severe; N (%) | | 3 (17) / 4 (22)/ 11 (61) |
| Hepatorenal syndrome; no/ type 1/ type 2 | | 15 (82)/ 2 (12)/ 1 (6) |
| History of variceal bleeding, no/ esophageal/ gastric | | 9 (50)/ 9 (50)/ 0 (0) |
| Hepatic encephalopathy, yes/ no; N (%) | | 3 (17) /15 (83) |
| Presence of ACLF (EASL-CLIF), yes/ no; N (%)  CLIF-C organ failure score, points 6/ 7/ 8  Type of organ failure, no/ kidney | | 5 (28)/ 13 (72)  13 (72)/ 3 (17)/ 2 (11)  13 (72)/ 5 (28) |

MELD: model of end-stage liver disease; TIPS: transjugular intrahepatic portosystemic stent shunt; INR: international normalized ratio; HRS: hepatorenal syndrome; sd: standard deviation; ACLF: acute on chronic liver failure; EASL: European association for the study of the liver; CLIF: Chronic liver failure consortium

.**SI-Table 3: Regression analysis of factors associated with hepatic vein levels of MCP-1.**

|  | **univariable model** | | **multivariable model** | |
| --- | --- | --- | --- | --- |
|  | **β** | **P-value** | **β** | **P-value** |
| Age | 1.69 | 0.18 |  |  |
| Creatinine (mg/dL) | -6.8 | 0.38 |  |  |
| Bilirubin (mg/dL) | -46.1 | 0.03 | -32.7 | 0.07 |
| Albumin (g/dL) | -47.2 | 0.002 | -39.5 | 0.002 |
| INR | 183.9 | 0.01 | 124 | 0.013 |
| Leucocytes (/nL) | 9.02 | 0.026 | 6.9 | 0.04 |

Multivariable linear regression analysis. Multiple correlations coefficient R= 0.89; Cohen’s d effect size f² = 3.815 ("high"); MCP-1: monocyte chemoattractant protein-1. N=17 patients.

**SI-Table 4: Regression analysis of factors associated with portal vein levels of MCP-1**

|  | **univariable model** | | **multivariable model** | |
| --- | --- | --- | --- | --- |
|  | **β** | **P-value** | **β** | **P-value** |
| Age | 1.8 | 0.23 |  |  |
| Creatinine (mg/dL) | -11.3 | 0.23 |  |  |
| Bilirubin (mg/dL) | -35.22 | 0.14 |  |  |
| Albumin (g/dL) | -51.83 | 0.004 | -42.6 | 0.003 |
| INR | 184.1 | 0.02 | 79.7 | 0.07 |
| Leucocytes (/nL) | 10.65 | 0.027 | 8.6 | 0.036 |

Multivariable linear regression analysis. Multiple correlations coefficient R= 0.87; Cohen’s d effect size f² = 3.056 ("high"); MCP-1: monocyte chemoattractant protein-1. N=17 patients.

**SI-Table 5: Differences of portal and hepatic vein levels of MCP-1 in dependency of disease complications.**

| **Levels of MCP-1**  **(pg/ml)** | **Portal vein** | | | **Hepatic vein** | | |
| --- | --- | --- | --- | --- | --- | --- |
|  | **Yes**  **(median)** | **No**  **(median)** | **P-value** | **Yes**  **(median)** | **No**  **(median)** | **P-value** |
| Hepatorenal syndrome | 50.1 | 44 | 0.47 | 68.2 | 51 | 0.41 |
| Gastrointestinal bleeding | 54.2 | 37.6 | 1 | 57 | 51.4 | 0.97 |
| Presence of ascites | 47.8 | 30.7 | 1 | 58.7 | 29.7 | 0.41 |

Mann-Whitney test; MCP-1: monocyte chemoattractant protein-1. N=18 patients.
